# Bladder pain syndrome/interstitial cystitis response to nerve blocks and trigger point injections

**DOI:** 10.1002/bco2.176

**Published:** 2022-06-18

**Authors:** Soha Patil, Gabrielle Daniel, Yogita Tailor, Marjorie Mamsaang, Janaki Natarajan, Erika Moody, Neha James, Rakhi Vyas, Allyson Shrikhande

**Affiliations:** ^1^ Pelvic Rehabilitation Medicine Clinical Research Foundation West Palm Beach FL USA; ^2^ The Feinstein Institute for Medical Research Northwell Health Manhasset NY USA

**Keywords:** central sensitisation, pelvic floor dysfunction, pelvic pain, peripheral sensitisation, trigger points

## Abstract

**Objectives:**

Bladder pain syndrome (BPS)/interstitial cystitis (IC) is a debilitating condition characterised by bladder/pelvic pain and pressure as well as persistent or recurrent urinary symptoms in the absence of an identifiable cause. It is hypothesised that in addition to organ specific visceral hypersensitivity, contributions of the hypertonic pelvic floor, peripheral sensitisation, and central sensitisation exacerbate this condition. The aim of this paper is to investigate outcomes of treating underlying neuromuscular dysfunction and neuro‐plastic mechanisms in BPS/IC patients.

**Methods:**

A retrospective chart review of 84 patients referred to an outpatient pelvic rehabilitation centre with a diagnosis of BPS/IC given to them by a urologist. All 84 patients failed to progress after completing 6 weeks of pelvic floor physical therapy and underwent an institutional review board approved protocol (IRB# 17‐0761) consisting of external ultrasound‐guided trigger point injections to the pelvic floor musculature, peripheral nerve blocks of the pudendal and posterior femoral cutaneous nerves and continued pelvic floor physical therapy once weekly for 6 weeks. Pelvic pain intensity and functionality were measured pretreatment and 3 months posttreatment using Visual Analogue Scale (VAS) and Functional Pelvic Pain Scale (FPPS).

**Results:**

Pretreatment, mean VAS was 6.23 ± 2.68 (95% CI 5.65 to 6.80). Posttreatment mean VAS was 3.90 ± 2.63 (95% CI 3.07–4.74). Mean FPPS before treatment was 11.98 ± 6.28 (95% CI 10.63 to 13.32). Posttreatment mean FPPS was 7.68 ± 5.73 (95% CI 6.45–8.90). Analysis of subcategories within FPPS indicated highest statistically significant improvement in the categories of bladder, intercourse and working.

**Conclusions:**

Analysis suggests the treatment was effective at ameliorating bladder pain and function including urinary urgency, frequency, and burning in BPS/IC patients.

## 1 INTRODUCTION

Bladder pain syndrome (BPS)/interstitial cystitis (IC) is defined by the International Continence Society as ‘Persistent or recurrent chronic pelvic pain, pressure or discomfort perceived to be related to the urinary bladder accompanied by at least one other urinary symptom such as an urgent need to void or urinary frequency’.^1^ Accurate nomenclature is still being developed; its aetiology is unknown and treatments are empirical and unsatisfactory. For the purposes of this paper, BPS/IC will be the constant nomenclature.

Prevalence approximations differ depending on procedures chosen to classify BPS/IC and diagnostic criteria. In older studies, prevalence of BPS/IC was comparatively rare (18.1/100 000 women and 10.6/100 000 men), and newer studies demonstrate a greater occurrence (52–197/100 000 women and 40–70/100 000 men) when physicians made the diagnosis. Estimates of patient self‐reports are much higher at 501–865/100 000 patients.^2^

Diagnosis is challenging because patients presenting with BPS/IC experience other unrelated disorders such as irritable bowel syndrome, fibromyalgia, chronic fatigue syndrome, and anxiety disorders in addition to their urinary bladder symptoms.^3^ Thus, selecting the appropriate treatment becomes difficult. Traditional treatments focus on the bladder, considering it as the source of pain and primary end organ. The multifaceted nature of BPS/IC however demands a systemic approach including non‐pharmacological treatment, conservative treatments, neuromodulators, and anti‐inflammatories to name a few.^4^ Non‐pharmacological treatments that utilise pelvic floor physical therapy and acupuncture to manage BPS/IC patients' hypertonic pelvic floor muscle dysfunction are traditionally recommended as first line treatments.^5^ Conservative treatments include behavioural and diet improvements, psychological distress management, and urogynecological exercises.^6^ Neuromodulators including tricyclic antidepressants alleviate bladder symptoms through their anticholinergic effects.^5^ Anti‐inflammatories like lidocaine control the pain and inflammation, allowing the neuropathic bladder to gradually return to its regular state by altering the neural pathways to prevent sending faulty signals.^7^

This investigation aims to establish the efficiency of an outpatient ultrasound‐guided peripheral nerve block and trigger‐point injection protocol aimed at treating the central neuro‐plastic mechanisms involved in BPS/IC known as (1) peripheral sensitisation and its associated neurogenic inflammation, (2) central sensitisation and (3) pelvic floor hypertonia.^8^ Central sensitisation results from increased membrane excitability and synaptic efficacy. This indicates neuro‐plastic changes in the central nervous system (CNS) and peripheral nervous system (PNS) in response to the inflammation, activity and potential neural injury seen in BPS/IC patients.^9^ Therefore, decreasing ectopic peripheral nociceptor activity and neurogenic inflammation with serial peripheral nerve blocks will ultimately decrease excessive peripheral neuronal input to the CNS and reverse the central sensitisation process. Pelvic floor hypertonia in BPS/IC patients causes neural ischemia around the peripheral pelvic nerves that contributes to the peripheral neurogenic sensitisation process.^10^ Therefore, it is essential to also address the pelvic floor hypertonia. This study was conducted to provide evidence for the efficacy of our outpatient neuromuscular protocol in treating patients with BPS/IC.

## 2 METHODS

### 2.1 Participants

Participants were 54 women and 30 men aged between 22 and 86 years diagnosed with BPS/IC by a urologist. The patients' chief complaints included perceived bladder symptoms with associated lower urinary tract symptoms such as bladder pain or pressure, pain or discomfort with bladder filling, urinary frequency, urgency and/or burning for at least 6 months. These patients came to an outpatient pelvic rehabilitation practice between the February 2020 and December 2020. All 84 patients undertook pretreatment evaluations with a detailed medical history and neuromuscular physical examination by one of 11 physiatrists. This examination evaluated the lumbosacral spine, hips and abdomen, as well as an internal evaluation of the pelvic floor, consisting of palpation of the levator ani sling to assess muscle strength and tone as well as palpation over Alcock's Canal and the ischial spines to observe tenderness/tingling known as Tinel's sign. Selected patients demonstrated either a Hypertonic Pelvic Floor with Myofascial trigger points or Allodynia along the pudendal nerve and its branches and along the adjacent posterior femoral cutaneous nerve bilaterally. Patients were excluded if they had an active infection, malignancy, persistent opioid use, incomplete or missing VAS or FPPS questionnaires or could not commit to 6 months of consistent pelvic floor physical therapy.

**FIGURE 1:**
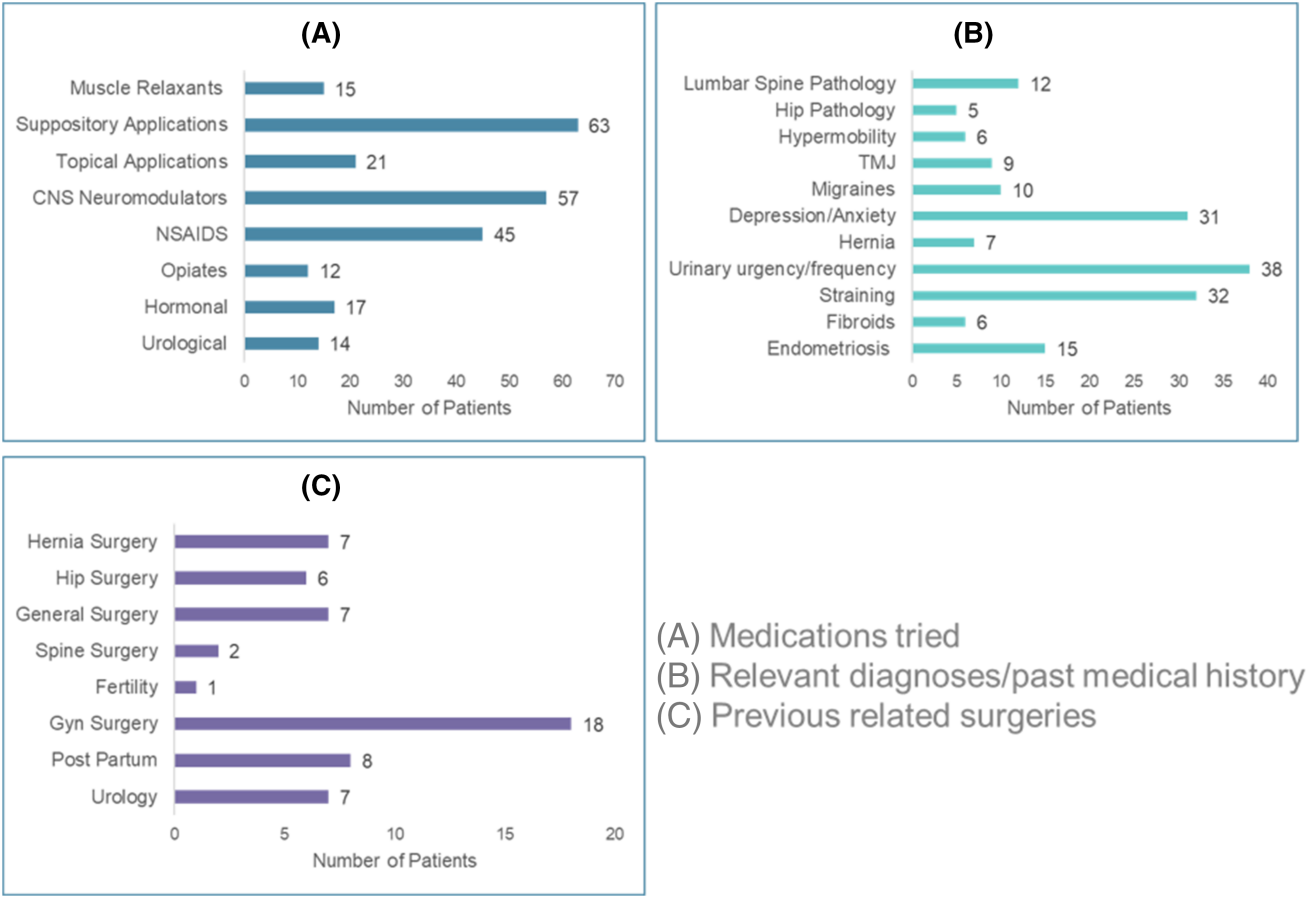
(A) Medications tried. (B) Relevant diagnoses/past medical history. (C) Previous surgeries of patients

Patient demographics are shown in Table 1, and previous medications tried, medical history and previous surgeries of all participants are in Figure 1.

**TABLE 1:** Patient demographics of patients with BPS/IC

Demographics table	
Participants (*n*)	84
Females (*n*)	54
Males (*n*)	30
Age (y) (mean ± SD) (min‐max)	41.19 ± 14.12 (22–86)
Average duration of pain (y) (mean ± SD) (min‐max)	5.33 ± 5.57 (0.5–23)

Abbreviation: BPS/IC, bladder pain syndrome/interstitial cystitis.

### 2.2 Procedures

**FIGURE 2:**
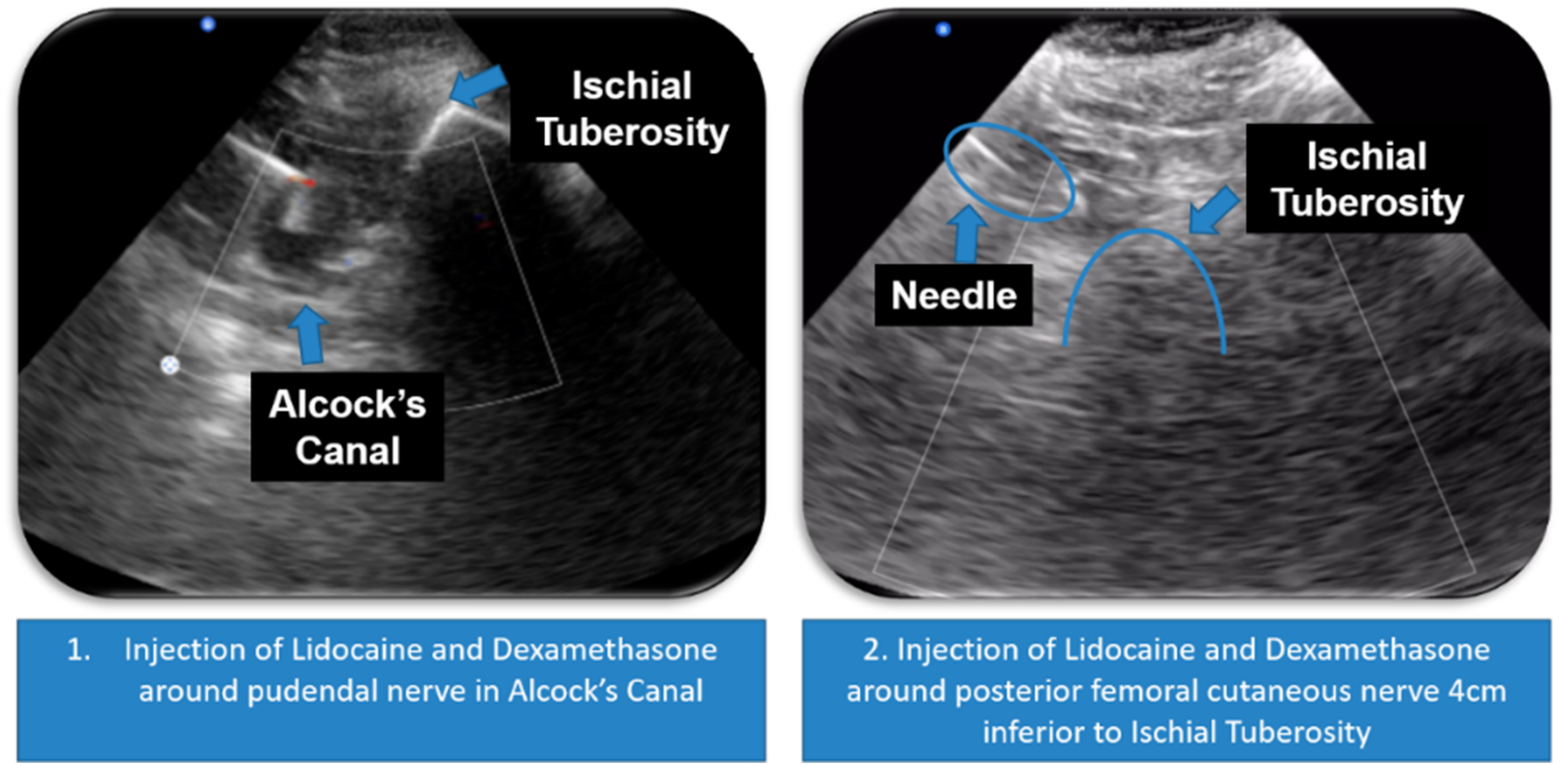
Ultrasound images of Alcocks Canal and Obturator Canal

A retrospective chart review was conducted for 84 patients referred to an outpatient pelvic rehabilitation centre with BPS/IC diagnosed by a urologist using the Pelvic Pain and Urgency/Frequency Symptom (PPUFS) Scale and O'Leary‐Sant Symptom Index. All 84 patients failed to progress after completing 6 weeks of pelvic floor physical therapy and underwent an institutional review board approved protocol (IRB# 17‐0761) consisting of external ultrasound‐guided trigger point injections using 1 cc of Lidocaine 1% to the pelvic floor muscular structure. Once weekly, for 6 weeks throughout the protocol, a global injection was administered into the iliococcygeus, pubococcygeus and puborectalis one side at a time.^11^ Therefore, each muscle of the levator ani sling was treated one time. With the patient lying in prone, a flexible 6‐inch, 27‐guage needle injects the targeted muscle from the subgluteal posterior approach, using an aseptic technique under ultrasound guidance. On ultrasound, myofascial trigger points are localised, stiff nodules that look like focal, hypoechoic regions with reduced vibration amplitude on vibration sonoelastography^12^ (Figure 2).

Simultaneously, while in the prone position, patients underwent ultrasound‐guided, peripheral nerve blocks of the pudendal nerve at Alcock's canal.^13^ Then, in supine position, patients underwent ultrasound‐guided peripheral nerve blocks of the posterior femoral cutaneous nerve at obturator canal at every visit, alternating right and left sides each time. For the first treatment, 2 ml of dexamethasone with 7 ml of 1% lidocaine was placed on each side, around each nerve. At subsequent appointments, the dexamethasone was replaced with normal saline for the peripheral nerve blocks. Regardless of the laterality of pain, this is an attempt to reduce peripheral neurogenic inflammation and attenuation of central sensitisation. For the entirety of the protocol, patients continued to attend pelvic floor physical therapy at facilities of their choosing. Pelvic floor physical therapy includes internal release of the pelvic floor hypertonic muscular structure, visceral mobilisation, scar tissue mobilisation, skin rolling along the lower abdomen and buttocks, nerve gliding along the pudendal and posterior femoral cutaneous nerves and diaphragmatic breathing.

The protocol was tolerated by all patients as it utilised a 27‐gauge needle with topical anaesthetic spray prior to the treatment. Patients were premedicated with diclofenac 75‐mg P.O. and returned to work the same day after sitting on ice for 10 min.

### 2.3 Statistical analysis

Data of patient's response to treatment were collected at their new patient consult and 3‐month follow‐up, with the 0 to 10 VAS to measure pelvic pain and FPPS to measure functionality. For VAS, patients rated their standard pain intensity across the past 24 h. Pelvic functionality on FPPS encompasses eight categories: bladder, intercourse, sleeping, walking, running, lifting, working and bowel. Patients ranked all categories from 0 to 4, showing regular function and serious debilitation, respectively. Experimenter bias was minimised by keeping follow‐up questions identical for all patients. Patients who had incomplete or missing VAS and/or FPPS questionnaires were excluded from this research study and not included in the analysis.

**FIGURE 3:**
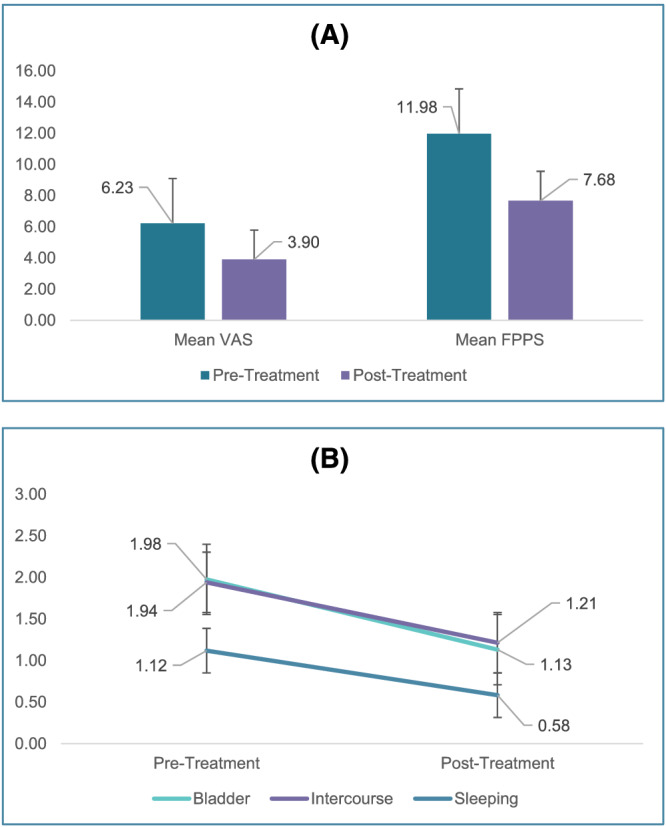
(A) Average Visual Analog Scale (VAS) and Functional Pelvic Pain Scale (FPPS) pre‐ and posttreatment. (B) Functional Pelvic Pain Scale (FPPS) pre‐ and posttreatment for most improved categories: Bladder, Intercourse, Sleep

The statistical significance between VAS and FPPS scores before and after our protocol was determined using a paired *t* test (Table 2) following a Shapiro–Wilks test for normality. Descriptive statistics data are presented as mean ± standard deviation with a 95% confidence interval. The sensitivity of our correlations is depicted via error bars in Figure 3.

**TABLE 2:** Visual Analogue Scale (VAS) and Functional Pelvic Pain Scale (FPPS) results table

	Pretreatment	Posttreatment	Difference	*P* value
VAS	6.23	3.90	2.33***	0.000
FPPS – TOTAL	11.98	7.68	4.30***	0.000
FPPS – BLADDER	1.98	1.13	0.85***	0.000
FPPS – INTERCOURSE	1.94	1.21	0.73***	0.000
FPPS – SLEEPING	1.12	0.58	0.54***	0.000
FPPS – WALKING	1.10	0.65	0.45***	0.000
FPPS – RUNNING	1.43	0.85	0.58**	0.002
FPPS – LIFTING	1.14	0.77	0.37**	0.014
FPPS – WORKING	2.01	1.43	0.58**	0.003
FPPS – BOWEL	1.26	1.05	0.21*	0.046

*** *P* < 0.001.

** *P* < 0.01.

* *P* < 0.05.

## 3 RESULTS

Eighty‐four patients underwent ultrasound‐guided, pelvic floor trigger point injections and peripheral nerve blocks; 41.1 ± 14.12 years was the average age of the 84 patients analysed, and 5.33 ± 5.58 years was the average period of pelvic pain. This is shown in Table 1. Statistically significant improvements were seen in all categories. Table 2 and Figure 3B summarise these results.

Before treatment, 6.23 ± 2.68 (*P* < 0.05, 95% CI = 5.65–6.80) was the average VAS score. After treatment, 3.90 ± 2.63 (*P* < 0.05, 95% CI = 3.07–4.74) was the average VAS score. Men saw a higher average decrease in VAS score. Average FPPS score prior to treatment was 11.98 ± 6.28 (*P* < 0.05, 95% CI = 10.63–13.32). Women saw a much higher decrease in FPPS total than men. Results differentiated by sex are summarised in Table 3. After treatment, average FPPS score decreased to 7.68 ± 5.73 (*P* < 0.05, 95% CI = 6.45–8.90). Evaluation of FPPS categories showed that improvement was statistically significant for bladder, intercourse and sleeping. For the bladder category, the average decrease in score after treatment was 0.85 (*P* < 0.05, 95% CI = 0.60–1.09) Before treatment, the average was 1.98 ± 1.05 (*P* < 0.05, 95% CI = 1.78–2.18). Posttreatment, the mean was 1.13 ± 0.93 (*P* < 0.05, 95% CI = 0.91–1.36). For the intercourse category, the average score decrease after treatment was 0.73 (*P* < 0.05, 95% CI = 0.41–1.04). Before treatment, the average was 1.94 ± 1.40 (*P* < 0.05, 95% CI = 1.62–2.26). After treatment, the average was 1.21 ± 1.48 (*P* < 0.05, 95% CI = 0.92–1.51). For the sleeping category, the average decrease in score post treatment was 0.54 (*P* < 0.05, 95% CI = 0.27–0.80). Before treatment, the average was 1.12 ± 0.91 (*P* < 0.05, 95% CI = 0.88–1.36). After treatment, the average was 0.91 ± 1.34 (*P* < 0.05, 95% CI = 0.39–0.78).

**TABLE 3:** Male and Female Visual Analogue Scale (VAS) and Functional Pelvic Pain Scale (FPPS) results table

	Pretreatment	Posttreatment	Difference	*P* value
VAS – TOTAL	6.23	3.90	2.33***	0.000
VAS – FEMALE	6.48	4.31	2.17***	0.000
VAS – MALE	5.80	3.17	2.63***	0.000
FPPS – TOTAL	11.98	7.68	4.30***	0.000
FPPS – FEMALE	12.91	8.15	4.76***	0.000
FPPS – MALE	10.3	7.07	3.23**	0.005
FPPS – BLADDER	1.98	1.13	0.85***	0.000
BLADDER – FEMALE	2.06	1.20	0.86***	0.001
BLADDER – MALE	1.83	1	0.83***	0.000
FPPS – INTERCOURSE	1.94	1.21	0.73***	0.000
INTERCOURSE – FEMALE	2.43	1.41	1.02***	0.000
INTERCOURSE – MALE	1.07	0.87	0.2*	0.02
FPPS – SLEEPING	1.12	0.58	0.54***	0.000
BLADDER – FEMALE	1.19	0.57	0.62***	0.002
BLADDER – MALE	1	0.6	0.4**	0.009

*** *P* < 0.001.

** *P* < 0.01.

* *P* < 0.05.

## 4 DISCUSSION

Because a sustainable and effective treatment of IC/BPS has yet to be established, there are a variety of medications and therapies used and/or studied by physicians. Botulinum Toxin A (BoNT‐A) has been studied via randomised trial for its effectiveness in treating symptoms of IC/BPS. However, there is not enough consistent data from these studies to establish a positive effect. Pentosan polysulfate sodium (PPS) is also a well‐documented treatment option and has been shown to successfully treat bladder pain, urinary urgency and frequency of micturition and thus an evident option for the treatment of IC/BPS symptoms. Therapies such as Mindfulness‐based stress reduction (MBSR) and Guided Imagery audio sessions have been studied via randomized trials as well, with MBSR demonstrating no change in VAS scores and Guided Imagery audio resulting in a decrease in VAS scores by 2.57, on average. Dimethyl sulfoxide (DMSO) has been studied via cohort studies and a single randomised trial. Treatment specificities are inconsistent and results from these studies vary greatly.^14^

Our investigation found statistically significant improvements in both pain and function for women and men aged 22–86 diagnosed with BPS/IC whose symptoms had not improved after completing 6 weeks of pelvic floor physical therapy. This supports the validity of an outpatient treatment protocol aimed at treating the underlying hypertonic pelvic floor myofascial pain and concomitant peripheral neurogenic inflammation often seen in the major pelvic nerves of BPS/IC patients. The pudendal nerve and the posterior femoral cutaneous nerves were treated as they are the two major sensory nerves that innervate the lower two‐thirds of the pelvis.^15^ Given the cross innervation between these two nerves, it is important to downregulate and reverse the aberrant firing and neurogenic inflammation that occurs in both nerves.^16^

BPS/IC is thought to have a major component of neurogenic inflammation and pelvic floor hypertonia that through the pelvic cross‐sensitisation process and inter‐neuronal connections, this causes injury/trauma to the bladder and results in symptoms commonly associated with BPS/IC.^8^ BPS/IC does not currently have a standard treatment algorithm. Our comprehensive outpatient treatment protocol addresses the pelvic cross‐sensitisation, neurogenic inflammation and pelvic floor hypertonia associated with BPS/IC.^17^

BPS/IC is traditionally correlated with chronic pelvic pain (CPP) and urinary urgency/frequency suggesting that these diagnoses and symptoms occur concomitantly. The colon and bladder, both involved in collecting, storing and excreting waste, are in immediacy and share mutual innervation from spinal afferent pathways.^11^ Studies^17^ demonstrating the bidirectional neural cross‐sensitisation of the bladder and colon support BPS/IC's neurogenic aetiology and could explain the overlap of BPS/IC with other CPP disorders.^8^ Therefore, a potential explanation for patients' responsiveness towards our injection protocol is desensitisation of the peripheral nerves in the overlapped pain patterns and treating the aberrant firing of the peripheral nociceptors in the distribution of the pudendal and posterior femoral cutaneous nerves.^18^

Decreasing peripheral neurogenic inflammation will ultimately break the spinal path‐way upregulation feedback loop from the PNS to the CNS and help to reverse the central sensitisation process that occurs in BPS/IC.^19^ Neurogenic inflammation is treated by (1) reversing the neural ischemia secondary to muscle compression, (2) using the potent anti‐inflammatory dexamethasone to deplete Substance P, a peptide released from sensory nerves during neuro‐genic inflammation^20^ one time on each side, and (3) repetitive exposure of the peripheral pelvic nerves to lidocaine, which has been shown to decrease neurogenic inflammation as it decreases the mast cell release of histamine.^21^

Hypertonic pelvic floor dysfunction has an estimated prevalence of 50–87% in BPS/IC patients.^22^ Trigger point injections to each muscle in the levator ani sling are used to reset the short, spastic and weak pelvic floor musculature. Treating the underlying hypertonic pelvic floor also helps create space for the nerves to flow with less impingement and aids in releasing the hyperirritable taut bands of muscle that contribute to promoting the chronic pain cycle. Active myofascial trigger points serve as a source of constant nociception contributing to the aberrant firing of peripheral nociceptors and ultimately central sensitisation.^12^ Alongside the protocol, patients continued pelvic floor physical therapy with the focus on lengthening their contracted musculature by releasing trigger points in the levator muscles and re‐cultivating their muscles to regular motion ranges.^22^ This contributes to the multimodal concept of the protocol; while physical therapy alone left patients with an incomplete resolution of their symptoms, once combined with other treatment modalities, improvements in pain and function were seen.

Multiple expert panels such as the American Urological Association,^7^ International Consultation on Incontinence,^23^ European Society for the Study of BPS (ESSIC)^24^ and European Association of Urology^2^ consider bladder pain and the existence of one other urinary symptom as diagnostic criteria for BPS/IC.^24^ Our study indicated the bladder category achieved the highest statistically significant improvement demonstrating the effect our protocol has on bladder pain and function in BPS/IC patients. A potential reason for this is the bladder neck no longer sits on a spastic pelvic floor preventing dysfunctional voiding caused by a hypertonic pelvic floor.^25^ In addition, there is noteworthy indication that afferent hyperexcitability due to neurogenic bladder inflammation and urothelial dysfunction is the source of pain sensation as the increased afferent activity demonstrates a major surge in the quantity of nerve fibres expressing substance P.^26^ The substance P expression may decrease as we address underlying neurogenic inflammation. The recurring contact to the anaesthetic lidocaine 1% in our protocol successfully downregulated bladder sensory nerves.^26^ Nocturia is one of the main symptoms that characterises BPS/IC,^26^ the bladder's downregulated sensory nerves keep urinary urgency and frequency in control, allowing uninterrupted sleep.

Pain with intercourse and a decreased sexual drive are common distressing characteristics women and men with BPS/IC as proven by the Multidisciplinary Approach to the Study of Chronic Pelvic Pain (MAPP) research network which examined the ‘most bothersome pelvic symptom’ in 191 men and 233 women with BPS/IC.^27^ Similarly, a survey by the Interstitial Cystitis Support Group concluded BPS/IC negatively affected the sex lives of more than 70% of patients.^28^ There are multiple etiologies of sexual dysfunction in BPS/IC patients, and the severity of pain has been noted to have a positive correlation with level of sexual dysfunction. This suggests that along with pan relief, sexual life improvement must be considered when treating patients with BPS/IC. Our protocol supports this ideology as demonstrated by the statistically substantial progress in our patients' capacity to return to pain free intercourse after undergoing treatment.

### 4.1 Limitations

A limitation of the study is the retrospective nature that prevents randomised control groups. The efficacy of our protocol in comparison with a placebo will not be possible as it would violate the ethics and trust of our patients who seek relief from their debilitating pain. A future improvement for outcome measures is to include the use of National Institutes of Health Chronic Prostatitis Symptom Index (NIH‐CPSI)^29^ and Female Sexual Function Index (FSFI) questionnaires to better understand the clinical significance of our results. Quantifying our patients' urinary symptoms, quality of life issues, sexual dysfunction and targeted pain at their new patient consult will help us analyse these improvements at their 3‐month consult. Another challenge is assessing the long‐term efficacy of our protocol, because flare ups can occur which may require further treatment beyond 3 months post‐protocol. Therefore, the next steps for patients beyond follow‐up visits consist of maintaining the progress of reduced pelvic pain and improved functionality that was achieved though the treatment protocol. Patients are given a neuromuscular re‐education home programme in combination with home internal dilator and/or wand work under the guidance of their physical therapist. In addition, self‐efficacy is promoted where patients are educated on lifestyle modifications to avoid flares up including exercise, diet, stress management and sleep.

## CONFLICT OF INTEREST

The authors declare no conflict of interest.

## AUTHOR CONTRIBUTIONS

A.S. developed the protocol and conceptualised this study. S.P. wrote the first draft of the manuscript and performed the statistical analysis. All authors were involved in data collection, editing, review and final approval of the manuscript.
